# Against Elective Forgiveness

**DOI:** 10.1007/s10677-018-9899-1

**Published:** 2018-05-28

**Authors:** Per-Erik Milam

**Affiliations:** 1 University of Twente, Enschede, Netherlands; 2 University of Gothenburg, Gothenburg, Sweden

**Keywords:** Forgiveness, Reasons, Blame, Elective, Moral requirement

## Abstract

It is often claimed both that forgiveness is elective and that forgiveness is something that we do for reasons. However, there is a tension between these two central claims about the nature of forgiveness. If forgiving is something one does for reasons, then, at least sometimes, those reasons may generate a requirement to forgive or withhold forgiveness. While not strictly inconsistent with electivity, the idea of required forgiveness strikes some as antithetical to the spirit of the concept. They argue that forgiveness is essentially elective. In this paper, I dispute these arguments. I argue that the intuitive plausibility of the position diminishes upon reflection and that the best arguments fail to explain why reasons to forgive, unlike most other reasons for action, cannot generate requirements.

It is often claimed—and often taken for granted—that forgiveness is elective. The idea here is that forgiveness is the forgiver’s prerogative, that it is rationally and morally permissible either to forgive or not to forgive. It is held to be an act of grace or generosity, a free gift, not something that can be deserved or owed.

It is also often claimed—and just as often taken for granted—that forgiveness is something that we do for reasons. We needn’t be aware of our reasons for forgiving, but in doing so we must be responding to reasons of some sort.^1^ And not just any reason will do. If I cease blaming you solely because I think you’re more likely to lend me money if I do—where my reason has nothing at all to do with your offence or character—then I have not forgiven. Likewise, while both forgiving and excusing involve ceasing to blame an offender, we distinguish them in virtue of the reason for doing so. We excuse because we recognise that the offender was not responsible while, in order to forgive, we must continue to believe that she was. Exactly what sorts of reasons count as reasons to forgive reasons is debatable, but, whatever the answer, it seems clear that forgiveness must be done for the right kind of reasons.

However, these two commitments are in tension with one another. The broader the offender’s discretion in forgiving, the weaker her reason needs to be to decide to forgive (or not). If forgiveness is strongly elective, then—so long as she does so for the right kind of reason—a victim can permissibly forgive or withhold forgiveness regardless of the reasons she has not to. But if we forgive for reasons, and if reasons can generate requirements, then I might sometimes be required to forgive. Certainly we think of most reasons as liable to generate requirements to act in some circumstances. Cheshire Calhoun states this worry clearly, saying that, “to the extent that repentance is allowed to count in favour of forgiving, to that extent the wrongdoer ceases to be viewed under a damning description, and forgiving ceases to be elective” (1992, 81).^2^ My aim is to resolve this tension. I suggest that the two commitments—to what I’ll call reasoned forgiveness and elective forgiveness—can be balanced, but that most previous attempts to do so have failed to recognise the depth of the tension between them. Some of those who have recognised it have proposed strongly elective accounts of forgiveness according to which the reasons one has cannot generate a requirement either to forgive or to withhold forgiveness (Calhoun 1992; Allais 2008, 2013). I reject this move and argue instead that a proper balance must take seriously the fact that forgiveness is reason-guided (Murphy 1982; Hieronymi 2001; Griswold 2007) and that this requires that we revise our view of why and to what extent forgiveness is elective.

Behind the view that forgiveness is elective lies a constellation of commitments. The core intuition is expressed in various ways: forgiveness is something to which no one has a right (Bovens 2009), is supererogatory (Gamlund 2010), is motivated by ‘inspiration’ as well as by reasons (Bell 2010), is an act of grace (Pettigrove 2012), and is essentially elective (Calhoun 1992; Allais 2013).^3^ My target is the claim that forgiveness is essentially elective and that our reasons to forgive can’t require that we do so. I argue that forgiveness is often elective but sometimes required—as you’d expect of any moral practice. Moreover, it will follow that, because it is a contingent fact which reasons one has, forgiveness can, in principle, be required for any offence, though severe offences will, in fact, require forgiveness rarely, if ever.

## Forgiveness

In order to evaluate arguments about elective forgiveness, we need to understand forgiveness generally. This section describes forgiveness in general terms and, while partisans will dispute the details, the arguments about elective forgiveness discussed in subsequent sections will not turn on disagreements about the picture of forgiveness sketched here.

Forgiving, as it is usually understood, has (at least) three conditions. In order to forgive, one must: i) experience a positive change in attitude toward a culpable offender, ii) about their offence, iii) for the right reasons. The first condition identifies forgiveness as a change of attitude. This has usually been understood as overcoming resentment (Murphy 1982; Hieronymi 2001) or blame more generally (Murphy 1998; Allais 2008; Bell 2013). However, a weaker version of the condition might require only that one overcome excessive blame (Westlund 2009; Garcia 2011) or forswear the desire to revenge or offset the offence (Zaibert 2009).^4^ The second condition captures the intentionality of forgiveness. In order to count as forgiveness, my change of attitude must be about the offence. For example, it is not enough that my attitude toward an inconsiderate coworker improves if that change is entirely due to my joy at having had a paper accepted at a prestigious journal. The same is true of blame. I am not blaming my coworker if my anger is prompted not by his inconsiderate behaviour, but solely by some act of cruelty I have just read about in the news. In each case, my change of attitude has nothing to do with the offender or the offence. The third condition captures the intuitive distinction between forgiving and other ways of ceasing to blame culpable offenders. Most philosophers who accept that we forgive for reasons also accept that some reasons are the wrong kind. On the one hand, if I come to view my inconsiderate coworker as just not worth it—not worth my anger, my effort, my care, or my regard—and let go of blame for this reason, I have not forgiven. This is not just a bad reason to forgive, it’s the wrong kind of reason. On the other, an apology, expression of remorse, or some other repudiation the offence seems to be a paradigmatic reason to forgive.

This third condition deserves more attention than I can give it here, but it suffices to note that most accounts of forgiveness, including my opponents in this paper, explicitly or implicitly endorse a right reasons requirement. While few accounts provide a list or a principled account of which reasons are reasons to forgive, many delineate the boundaries of forgiveness by identifying various wrong kinds of reason (Hieronymi 2001, 530; Allais 2008, 43–44 fn.26; Garrard and McNaughton 2010, 114). For example, one who tries to overcome his resentment solely because the stress is causing him to lose sleep has not forgiven (Richards 1988, 79). An exception is Jeffrie Murphy, who lists five general reasons that he argues are of the right kind, including repentance and having had good intentions (1982, 508–509). While his list has been disputed (McGary 1989; Griswold 2007; Pettigrove 2012), most critics accept some version of the right reasons requirement.^5^

At first glance, this may seem contrary to common usage, but we actually acknowledge a variety of ways in which victims overcome blame without forgiving. Imagine a mistreated employee who ceases to blame their supervisor solely because they hope that doing so will make the supervisor more likely to recommend them for a promotion. This may be a prudent thing to do, but it seems natural to deny that this employee has forgiven. In this case, like Richards’ case above, the reason for overcoming blame has nothing to do with the offender or the offence; the victim would have the same reason to overcome their blame if the offender was excused. In short, the reason seems to support some other practice than forgiveness.^6^ Nor is the distinction being drawn here between forgiveness and non-forgiveness merely in the service of an ideal theory. What the right reasons requirement acknowledges is precisely the “messy details” of our forgiveness practice (MacLachlan 2009, 187). It recognises that one is doing something importantly different when ceases to blame because one perceives that moral realignment or repair is possible and when one does so because, despite the fact that realignment is not possible (or precisely because it’s not), one’s psychological well-being is in one’s own hands.

## Elective Forgiveness

In this section, I distinguish different ways of understanding the commitment to electivity in order to identify my particular target and to illuminate the ways in which philosophers have attempted to balance elective and reasoned forgiveness. The core commitment is that it’s up to the potential forgiver whether or not to forgive. One might then say either that it just so happens always to be elective or that there is something in the nature of forgiveness such that it must be. For either view, but especially the latter, the question is what makes forgiveness elective and why it’s essential. My target is the following claim:

*Essentially Elective Forgiveness (EEF)*: it is necessarily morally and rationally permissible for one either to forgive or not to forgive an offence.

This is a widely held view. However, to my knowledge, Cheshire Calhoun (1992) and Lucy Allais (2008, 2013) are among the few philosophers to have argued for it explicitly.^7^ Others have discussed the electivity of forgiveness, but almost all of them shy away from EEF in some way or other. Examining these other accounts will illuminate the intuitive case for EEF as well as its limitations, so I begin with this task.

The claim that it’s wrong for an offender to demand forgiveness from their victim does not itself imply EEF. Pamela Hieronymi and Margaret Urban Walker defend this weaker claim on the grounds that forgiveness is a significant burden to impose on a person who, by hypothesis, has already been victimised (Hieronymi 2001, 552; Walker 2006, 178–179). Rather, the relevant considerations are the psychological difficulty of forgiving and respect for one on whom this difficulty is imposed against her will. Likewise, we can accept that it is sometimes (probably often) inappropriate to pressure victims to forgive, even when they are morally required to do so. It may be inappropriate to pressure a grieving spouse into moving on even if they owe it to their children to do so. Other ways of engaging with such agents is more effective and more appropriate, especially if they occupy a vulnerable or difficult position. Moreover, this is true even if we accept that anger and blame are an important part of our responsibility practice (Strawson 1962; Murphy 1982; Hieronymi 2001), and even if we take attitudes like resentment and contempt to be appropriate in a broader range of circumstances than traditional apologists of blame have acknowledged (MacLachlan 2010; Bell 2013). These accounts do not appeal to anything essential to forgiveness and they allow that forgiveness is not always optional.

Luc Bovens (2009) argues that an offender cannot have a claim right to forgiveness, but allows that it can still be wrong not to forgive. He explains the electivity of forgiveness by reference to a distinctive feature of rights that plausibly precludes a right to forgiveness. On this account, by committing an offence the offender “squanders” her right to others’ respect for her status as an equal member of the moral community. To forgive is to commit oneself to once again respect the offender’s equal status, but whether to make this commitment is up to the victim (2009, 231–232). The offender can give him reasons, and, while she cannot demand and is not owed a favourable decision, the reasons themselves might require that he forgive her. Likewise, Espen Gamlund argues that forgiveness is supererogatory, but accepts that circumstances can conspire to create an all things considered duty to forgive (2010, 553). His reasoning seems to be that any plausible normative ethics must allow for such cases and that forgiveness is not different from other practices that are typically, but not essentially, supererogatory. Bovens and Gamlund both attempt to explain and motivate electivity, but both conclude that moral reasons can be such as to require or prohibit forgiveness.

Macalaster Bell (2010) addresses the tension between the two commitments more directly. She accepts that reparative acts—e.g., expressions of remorse, repentance, and apology—create reasons to forgive and she acknowledges that such reasons can generate requirements. However, she argues that in order for the victim to appreciate those reasons, a reparative act must also be “inspiring”. It must make those reasons salient and meaningful to the potential forgiver and invite a response from her (2010, 211). Forgiveness is elective when (and because) the reparative act was not inspiring and the victim has not been led to appreciate the reasons it offers to forgive. It would be unreasonable to expect a person to forgive on the basis of reasons she has not been inspired to appreciate. However, one can be morally and rationally required to forgive if a reparative act creates strong reasons to do so and inspires appreciation for those reasons (2010, 213).

Glen Pettigrove (2012) describes forgiveness as an act of grace or unmerited beneficence. His account of the nature of forgiveness is also directly motivated by the tension between the two commitments, but he too shies away from the strong position espoused by proponents of EEF. He explains gracious forgiveness as an act of unmerited favour, but he too allows that some cases of forgiveness are not acts of grace and that we sometimes forgive because we believe it to be deserved (2012, 152). While one could imagine a view according to which forgiveness is essentially gracious, Pettigrove seems to accept the weaker claim that gracious forgiveness is only one type.^8^

Each of these views attempts to capture the electivity of forgiveness while accepting that reasons can generate requirements to forgive or not forgive. So we might not seem to be lacking for opposition to EEF. However, while these accounts of elective forgiveness converge on the point of tension, each is vulnerable to the arguments made by proponents of EEF because all of them take for granted that reasons to forgive can generate requirements. But this is precisely what arguments for EEF deny. My aim in what follows is to disarm those arguments and offer a unified account of reasoned forgiveness that captures both the reservations voiced by these moderate positions and the intuitive pull of elective forgiveness.

## Counterexamples

There are three ways one might try to support the view that forgiveness is essentially elective: by appeal to the nature of forgiveness, by appeal to its norms, and by appeal to its intuitive plausibility. In the next section, I argue that its nature and norms allow for moral and rational requirements to forgive. In this section, I try to undermine the intuitive plausibility of EEF by showing that it’s both conceivable and plausible that some victims would (and do) do wrong either by forgiving or not forgiving. In doing so, I focus on cases of everyday wrongdoing. If we wish to understand forgiveness, we must examine our own experiences with it. Fortunately, while few can avoid ever being wronged, most of us will be spared the horrific experience of violent assault or the murder of a loved one. The claim that forgiving is sometimes required or impermissible may seem implausible if we focus only on murder, slavery, and genocide. We must not ignore such horrors, and a good account of forgiveness will illuminate these cases too, but these egregious wrongs should not disproportionately shape our understanding of the ubiquitous experience of forgiving. With this in mind, consider two cases.

- *Case 1.* Peggy’s coworker Al often acts disrespectfully toward her; on the worst occasions he belittles and humiliates her.

- *Case 2.* Danny promises to pick up Jesse’s twins from daycare but forgets to do it. The daycare calls Jesse who has to miss an important job interview in order to pick them up.

You can fill in the details of these cases as you wish, with the following restrictions. Peggy has some reason to forgive Al—suppose he has apologised. And Jesse has some reason not to forgive Danny—at least the fact of his culpable wrongdoing. These reasons not withstanding, it seems to me that there are some possible combinations of the offender’s attitudes and actions, the victim’s psychology, their respective histories, and the present circumstances and likely consequences of forgiving (or not) such that it would be wrong for Peggy to forgive and for Jesse not to forgive. (To be clear, though, there will be many cases in which it would not be wrong for Peggy to forgive or for Danny to withhold forgiveness.) Again, you can imagine the cases as you wish, but we might flesh them out as follows:

- *Case 1.* Peggy is always upset by Al’s mistreatment. Often this takes the form of anxiety and sadness, other times contempt, and sometimes anger and retaliation. Al usually apologises and she has always forgiven in the past, but the pattern of abuse has not changed. Moreover, Peggy has begun to realise that these apologies are the only reason she has to forgive him, while the rest of his behaviour speaks against forgiving. None of his other actions show remorse and they are often accompanied by veiled threats, signs that he feels entitled to treat her this way, and suggestions that he will reward her professionally if she puts up with his harassment. At best he appears to have convinced himself that his abuse doesn’t reflect his true feelings. Peggy has also begun to worry that, by forgiving, she is encouraging further mistreatment and implying to others that she condones his behaviour. Finally, she knows from speaking with others in the office that they share her concerns and believe that her willingness to forgive is reckless.

- *Case 2.* Jesse is upset with Danny, but Danny apologises, shows Jesse that he feels terrible, and promises never to be so thoughtless again. There is still some reason for Jesse not to forgive, but nothing beyond the fact of the wrongdoing. The twins haven’t been harmed in any serious way, Danny’s remorse is sincere, Jesse recognises this, and, moreover, Danny has done everything Jesse could reasonably ask to regain his trust. Moreover, Danny’s relationship with Jesse and the twins is something very important to him (and them) and, by refusing to forgive, Jesse is damaging a valuable relationship with little cause. Finally, in calmer moments Jesse suspects that his unwillingness to forgive is motivated, in part, by unwarranted anger about a political disagreement he and Danny had a few weeks back.

Of course, even if an action (or omission) is morally wrong, it’s not thereby irrational. However, I think that on some elaborations of the above cases it would also be rationally impermissible to forgive or not forgive. Imagine, for example, that Jesse acknowledges that there is nothing else he can reasonably ask of Danny or that Peggy has reason to doubt the sincerity of Al’s apologies and other reparative acts.

If you were able to imagine compelling examples of impermissible and required forgiveness, or if you found my elaborations compelling, then you already have some reason to reject EEF. If forgiveness is even sometimes morally or rationally required, then it cannot be essentially elective. But if not, you’re probably not alone. Reasons may strongly favour forgiving without requiring it. There may be something about the nature or norms of forgiveness that makes it essentially elective. In the next section, I support my intuition that forgiveness is not always optional with arguments. I consider and contest arguments for EEF and raise additional independent objections to the view.

## Essentially Elective Forgiveness

Essentially elective forgiveness is in tension with a natural understanding of forgiveness as a reason-guided practice. In order to forgive one must have the right kind of reasons. One can have reasons for or against forgiving, or both. And these reasons can add up, giving one more or less reason to forgive, all things considered. But, if reasons for and against forgiving can add up, then sometimes the weight of one’s reasons can generate a requirement either to forgive or not. This is how reasons, especially moral reasons, typically work. I generally have reasons not to go to the pub after work. Those reasons rarely generate a requirement not to, but they easily could—e.g. perhaps I’ve been drinking too much recently, or my partner is sick at home, or we need to save money. There are combinations of reasons that might render an otherwise innocent trip to the pub morally or rationally impermissible. Forgiving seems to be the same.

This is true even if we grant the potential forgiver some manner of prerogative—e.g., a prerogative to act contrary to what she has all things considered most reason to do (Scheffler 1992).^9^ Again, we accept this conclusion for other practices. I might have reason not to excuse some misconduct, but sufficient reason otherwise that not doing so is impermissible. For example, I might have reason to doubt that clinical depression can seriously undermine a person’s agential capacities, but nonetheless have overwhelming reason to think it can and therefore that it can excuse. Why should we think that forgiveness is any different?^10^ Some philosophers have recognised the force of this challenge and tried to avoid its conclusion in different ways, but I will argue that their attempts are unsuccessful.

Proponents of EEF must conceive of forgiveness as fundamentally different from other practices, like excusing and justifying, whereby we cease to blame in response to reasons. These practices are not essentially elective. So, to avoid being arbitrary, the defender of EEF must explain how forgiveness is unique amongst our ceasing-to-blame practices in being, in principle, undeserved. Brandon Warmke (2015) gives one argument for thinking that forgiveness might be a special case. If an apology or some other gesture can generate a requirement to forgive, then the wrongdoer can oblige a victim to forgive. And, since receiving forgiveness is a benefit, it follows that a wrongdoer can oblige a victim to benefit him. But, Warmke assumes, a wrongdoer cannot oblige a victim to benefit him, and so cannot oblige a victim to forgive him. He concludes that we should accept that some gestures give reasons to forgive, but not requiring reasons (2015, 505–506). However, this seems to me the wrong lesson to draw. The purpose of apologies and other gestures of remorse is precisely to give your victim reasons to forgive—and, yes, to benefit you by forgiving. And if a wrongdoer can give his victim reasons to forgive, then he can give her requiring reasons to forgive—unless there is something about reasons to forgive that makes them essentially non-requiring. We still need an argument for this claim.

### Kinds of Reasons

One strategy is to argue that reasons to forgive are normative reasons of a particular type and that reasons of this type cannot generate requirements. It seems intuitive that reasons can render some behaviour appropriate but not obligatory. For example, a stranger’s questionable parenting technique might make it appropriate for me to suggest a different strategy for responding to misbehaviour, but I am not thereby obliged to make this suggestion. The distinction between justifying and requiring reasons might seem precisely what’s needed for this kind of argument. Perhaps reasons to forgive have justifying force rather than requiring force; perhaps they can generate permissions but not requirements (Gert 2007). If reasons to forgive are essentially justifying reasons and justifying reasons cannot generate requirements, then reasons to forgive cannot generate a requirement to forgive (or not forgive).

However, this argument fails. We can accept the distinction between justifying and requiring reasons, but also note that a reason may have mere justifying force in one circumstance but requiring force in another (2007, 542). For example, given my present income, the fact that I can prevent a child from dying of a preventable disease might justify giving $1000 to a relevant charity. But if I’m a billionaire, the very same reason plausibly requires that I give that money, all else being equal. Likewise, while I am not required to question the stranger about their parenting, I may be required to do so with a friend, a sibling, or my partner. The same would seem to be true of forgiveness, unless reasons to forgive cannot generate requirements—i.e. unless they can only ever generate permissions. Some reasons are arguably like this. For example, self-defense may justify but never require killing one’s attacker (Gert 2007, 539). However, even those who distinguish justifying and requiring reasons, do not claim that these are two types of reason. Rather they are distinct “roles” that a reason can have (Gert 2007, 542; Greenspan 2010, 190–191).

Moreover, even if we accept that some reasons are essentially justifying, reasons to forgive do not seem to be reasons of this sort. For example, given the close connection between forgiving and blaming, they seem more like reasons to punish than reasons to kill in self-defense. Having committed a crime often justifies punishment without requiring it—e.g., mercy is usually an option. But often it does require punishment—e.g., when failing to do so would severely undermine the deterrent effect of the law. Generally, it seems more plausible that forgiveness is frequently permissible because one typically has some good reason to forgive (or not) rather than because reasons to forgive (or not) cannot, by their nature, generate a requirement in the other direction.

This is not meant to be a decisive argument against the claim that reasons to forgive are essentially justifying. My aim has rather to been to demonstrate that further argument is needed to show that some types of reasons cannot generate requirements and that reasons to forgive are of this type. As it stands, it is not clear that justifying reasons are a type at all, much less that reasons to forgive belong to this type. Thus, the burden of proof remains on the proponent of EEF and the challenge stands. In virtue of what, if anything, are reasons to forgive unable to generate moral or rational requirements?

### Division and Revision

Another strategy available to defenders of EEF is to try to avoid this challenge by denying that forgiveness is reason-guided in the way we imagine. These philosophers accept the threat posed by reasoned forgiveness, but argue that standard conceptions have misunderstood the basic function and significance of our practice of forgiving. Call this the revisionist strategy. For example, Cheshire Calhoun (1992) defends EEF by drawing a distinction between what she calls minimalist forgiveness (MF) and aspirational forgiveness (AF). Minimalist forgiveness is incompatible with EEF because it is something we do for reasons and those reasons can require and prohibit forgiving. But Calhoun argues that MF is actually a form of excuse. It’s a matter of separating the offender from her offence (1992, 83). We forgive in the minimalist sense when we recognise that the offender is no longer tied to her action in the right way—e.g., because she’s had a change of heart (1992, 88). However, whereas MF requires a perceived change in the offender as the ground for forgiveness, AF does not try to separate the offender from the offence in any way. Contrary to our traditional conception (Murphy 1982, 509), AF does not require that the person be different than she is. Rather, it requires that one try to understand how the offence makes sense within the context of the offender’s life and experience—e.g., perhaps considerations of integrity outweighed moral concerns when deciding what to do.

Aspirational forgiveness is essentially elective (1992, 95–96).^11^ First of all, the kind of sympathetic engagement with one’s offender necessary for AF is too demanding to be required. Second, AF requires that the potential forgiver not demand, or even attempt to secure, moral realignment from the offender. It requires that one accept that an offender’s actions might make sense for her even if they don’t make moral sense. But this cannot be required either. A victim cannot be required to accept being wronged even for compelling non-moral reasons. In short, AF better captures what we mean by forgiveness than MF, which is actually a form of excuse, and AF is essentially elective insofar as the necessary conditions on doing it cannot be demanded of a victim.

Despite its subtlety and insight, however, Calhoun’s account does not succeed. First, she is committed to the claim that AF is the only genuine form of forgiveness, but AF fails to capture much of our experience of forgiving. Forgiveness sometimes looks like this: Salman forgives Emily for telling an offensive joke because she apologises and expresses shame and regret at having been thoughtless and insensitive. Forgiveness is sometimes—I’d guess typically—a response to the perceived moral realignment of offender and victim in the direction demanded by the victim’s blame. But this practice is not aspirational and is not essentially elective in the way Calhoun takes AF to be, so her view cannot count it as forgiveness. Of course, we could accept AF and its implication that forgiveness is rarer than we think, but, while I suspect that we forgive less often than our praise for forgiveness would suggest, the fact is that AF fails to capture even the most paradigmatic cases of forgiveness. (This may be why Pettigrove allows that not all forgiveness is gracious forgiveness.) This worry might be thought to beg the question since Calhoun is clearly offering a revisionist conception of what it means to forgive and might accept that her account fails to capture paradigmatic cases. However, while we should acknowledge that the phenomena she describes is a genuine part of our moral life, it does not have better claim to the label ‘forgiveness’ than MF or the various other conceptions on the market.

On the contrary, while Calhoun argues that MF resembles excuse more than forgiveness—because it suggests that the offence does not attach to the offender, just as uncontrolled or non-culpably ignorant actions do not—I would make a similar charge against AF. Coming to understand an offender’s reasons for acting in accordance with, e.g., her sense of integrity rather than the demands of morality, and ceasing to blame on that basis, looks like a sort of non-moral justification rather than forgiveness. By allowing the offender to live and act as she will, the purported forgiver has accepted—or perhaps condoned—her decision to privilege non-moral considerations over the demands of morality. Indeed, Calhoun herself states that her account requires a commitment to “deprioritising the moral” in our conception of forgiveness (1992, 92). She does not suggest that potential forgivers accept mistreatment as morally justified, but rather that we recognise that one can act wrongly but for recognisably good reasons, as when one takes non-moral values to outweigh moral demands (Wolf 1982). Again, though, we can acknowledge this practice but deny that the phenomenon is forgiveness. If Salman realises that Emily told the offensive joke because she took its structure and other aesthetic features to outweigh the moral reasons for not telling it, then, if he accepts her assessment, he has no reason to forgive. In telling the joke, she showed what he takes to be sufficient regard, all things considered. But if he doesn’t accept the assessment, then it doesn’t make sense for him to forgive unless she gives him some reason to think she won’t keep telling offensive jokes. He needs some evidence that their values are aligned.

This might look like bickering over a label, but it’s not. Our understanding of forgiveness is bound up with other elements of our responsibility practice, including blame, the reactive attitudes, and our perceptions of others’ quality of will (Strawson 1962, 152–153). But the account of AF obscures the connection between forgiveness and blame. The main purpose of blame is to motivate a moral realignment between offender and forgiver, but this is incompatible with AF, so blame for this reason is unjustified.^12^ For Calhoun, blame is something to be overcome, not because moral realignment has been achieved, but because trying to achieve it is antithetical to AF. A forgiver must overcome her blame in order to forgive, but forgiving is actually something else entirely.

Finally, even if we were to accept that AF is the only form of forgiveness, it still seems implausible to say that, in principle, morality cannot demand the kind of sympathetic engagement that AF requires of a victim. Calhoun has replaced the kind of reasons for which we forgive—reasons of understanding rather than reasons of repentance—but she’s not given an argument for why these reasons can’t generate requirements. This is still the challenge facing the defender of EEF.

### Reasons to Feel

A third strategy is to meet the challenge head on and argue that reasons to forgive cannot generate requirements. Lucy Allais (2008, 2013) makes an argument of this sort. She accepts the traditional view of forgiveness as overcoming blaming attitudes and separating the offender from her offence and instead targets the claim that reasons to forgive can oblige one to forgive. Her argument can be understood as follows:

1. Reasons to forgive are reasons to not feel a blaming attitude.
2. Reasons to feel can’t generate requirements.

Therefore,

3. Reasons to Forgive can’t Generate Requirements

Following Peter Goldie, Allais motivates (2) by suggesting that, “Affective attitudes can be made intelligible, appropriate or fitting by the evidence, without its being the case that they are rationally or epistemically mandated” (2013, 646; Goldie 2002, 78). So, while the evidence currently mandates that I believe it’s a sunny autumn day, and while that evidence might make gratitude fitting, I am not rationally obliged to feel grateful, even if I have no reason not to. Allais applies this idea to resentment and forgiveness:

You are entitled to allow the way you affectively see someone to be informed by her actions. Even if a person has distanced herself from the wrong, this need not oblige you to change the way you see her. She still did it and that fact that she is sorry does not change this, and does not, generally, entitle her to demand that you see her in a way which is not informed by this action. On the other hand, even if a person has not done anything to undo the warrant for resentment which is given by her wrongdoing, you have the option of affectively seeing her in a way which is better than the wrongdoing warrants (647).

This account of affective attitudes appears to fit with the norms of resentment. While wrongdoing entitles one to resentment, it does not require it. Someone who fails to resent her offender, or chooses not to, does not make a moral or epistemic mistake. And the same is true for forgiveness.

Before evaluating this argument, note that Allais’ defense of EEF only succeeds if we accept that blame and forgiveness are essentially affective. If forgiveness is a matter of forswearing blame, but blame does not require a hard feeling, then the fact that one can’t be required to forswear this feeling is irrelevant. Proponents of EEF who deny that forgiveness is fundamentally about overcoming reactive attitudes would do well to keep this point in mind. Allais’ argument is not available to someone like Warmke (2016a, b) or Cornell (2017), for example. However, I’m sympathetic to Allais’ conception of blame as requiring hard feelings, as well as to her conception of forgiveness as overcoming these feelings, so I assess her argument on its own terms.

First, while it’s plausible that one can’t be required to feel a particular way, this seems to be largely because we can’t always control how we feel. In cases where how one feels is voluntary, the claim that one might be required to feel a particular way seems more plausible. For example, when my mom tells me not to feel sad that my grandmother has died because she wanted to die and to die on her own terms, she is not only giving me a reason not to feel sad, she’s also counting on my gaining a measure of control over my sadness. She’s knows that I’ll be (better) able to eventually overcome my sadness when I realise that my grandmother’s death was, all things considered, a good thing. This shows how strong premise 2 really is. It implies that one cannot be required to feel a particular way even when one has a good reason and can control whether one does.

Second, consider the analogy between forgiveness and resentment, which is also essentially elective on this view. I contend that resentment is not always elective. Reasons to feel can license criticism of our feelings. Fear and disgust can both be morally and rationally prohibited. Insofar as we are responsible agents with (some) control over our feelings—and acknowledging that sometimes we aren’t—it’s wrong to fear others in virtue of their race or religion and irrational to fear unlikely dangers. Likewise, it’s wrong to be disgusted by transgender people or homosexual intercourse and irrational to be disgusted by food because it has a strange-sounding name, like yoghurt. Nor is resentment an exception to this rule; it follows the same pattern. One can be morally or rationally required not to feel, or to overcome, resentment when one rejects the source of the feeling—e.g., if you recognise that it’s driven by avoidable prejudice.

Or consider the main function of blame. On some accounts of blame, including some that are otherwise consistent with Allais’ commitments, the main point and purpose of blaming attitudes like resentment, contempt, and guilt is to object to mistreatment, protest misconduct, and demand moral realignment (Bell 2012; Fricker 2016). But if this is the main purpose of such attitudes, then there will be appropriate and inappropriate times for them. In some cases, blaming attitudes will be conceptually precluded—e.g., blame simply isn’t ‘on the table’ if one believes the person hasn’t committed an offence. But we’re not concerned with such cases here. In other cases, though, reactive attitudes will be possible but inappropriate. For example, radically disproportionate but otherwise fitting anger and blame is wrongful and can be rationally impermissible, too. Forgiveness can be disproportionate in the same way—e.g., when one forgives a grave offence in response to a pro forma apology or a vague sign of regret. It seems then that inappropriate resentment and dysfunctional forgiveness can plausibly be viewed as morally or rationally impermissible in some cases.

At the very least, even if reasons to feel do not generate requirements on their own, one might nonetheless be required not to feel a particular way when doing so is contradicted by what one justifiably believes. For example, while Jesse may not be required to forgive (or cease to resent) Danny just because he has most reason to cease resenting, he may nonetheless be required to forgive if his reason to continue resenting is at odds with his justified belief that there is no remaining reason to resent Danny beyond the fact of his offence. What we have reason to believe does sometimes make demands on and license criticism of our feelings.

Finally, even if we reject her argument, Allais’ reasoning lends itself to a slightly different defense of EEF. Rather than argue that reasons to feel cannot generate requirements, one might argue instead that *some* reasons to feel cannot generate requirements and that reasons to forgive are of this type. In particular, one might claim that reasons to forgive are reasons to love. This suggests the following argument: reasons to forgive are reasons to love; reasons to love cannot generate requirements; therefore, reasons to forgive cannot generate requirements.

However, while it appears plausible on its face, this defense also fails to support EEF. Consider each of the premises. First, are reasons to forgive really reasons to love? If we mean something like romantic love or close friendship, then no. A reason to forgive a person may sometimes be a reason to love them, but not always. A partner’s remorseful admission of infidelity might be a reason both to forgive and to keep loving them, but a coworker’s apology for their workplace harassment is usually not, by itself, a reason to love them in this sense. But suppose that we mean something like a general benevolent attitude. For example, Garrard and McNaughton suggest that we have reason to love everyone in this way and that this is a reason everyone has to forgive unconditionally (2010, 120). However, even if all reasons to forgive are reasons for general benevolence, this kind of love can be required. Ernesto Garcia ascribes to Bishop Butler the view that, fundamentally, to forgive is to avoid both excessive and deficient resentment toward our wrongdoers and that doing so requires that we feel and express “impartial goodwill and civility” toward all wrongdoers (2011, 17). Some, myself included, would reject this as a plausible account of the nature of forgiveness, but the claim that all human beings our deserve impartial good will or a basic level of regard is eminently plausible.

Thus, the proposed adaptation of Allais’ argument ultimately succumbs to a dilemma. We can understand love as either romantic love or general benevolence. Reasons to forgive may be reasons to feel and express a general benevolence, but these reasons plausibly generate requirements. Reasons for romantic love may not generate requirements, but reasons to forgive are not reasons of this sort. For neither type of love is it true both that reasons to forgive are reasons to love and that reasons to love cannot generate requirements.^13^

### Forgiveness and Generosity

Neither of these strategies—reconceiving of forgiveness as aspirational or arguing that reasons to forgive are reasons to feel—adequately demonstrates that reasons to forgive cannot, in some cases, generate a requirement to forgive or not forgive. In the remainder of this paper, let me briefly consider one final argument for EEF.

One might draw an analogy between generosity and forgiveness (Pettigrove 2012; Allais 2013). If generosity just is giving more than is deserved, then the following argument is tempting: forgiveness is an act of generosity, acts of generosity are essentially elective, so forgiveness is essentially elective. (Though one might still be required to develop traits of generosity and forgivingness.) However, while the analogy is illuminating, it does not necessarily support EEF. We might conceive of forgiveness as a kind of charity, which is a form of generosity but also sometimes morally required. Thus, even if forgiveness is like generosity, generosity—in the form of charity—can be required. One might object that not all acts of charity are acts of generosity. One could argue that the concept of charity has changed and that charity no longer implies generosity, or that we have discovered that some acts of charity are not acts of generosity as we previously supposed. However, we can make the same kinds of claims about forgiveness in either case.

The generosity argument does get something right, though. Forgiveness is helpfully understood as a virtue. But the analogy (or identification) with generosity fails to capture both the normative role of forgiveness in our lives and our lived experience of forgiving. Howard McGary argues that we should think of forgiveness as similar to courage, something we hope people will do but don’t expect or require (1989, 350). Indeed, he objects to the claim that forgiveness can be required precisely because it implies that forgiveness is a virtue like honesty, which can be required, rather than like courage. But this conception of the virtue of forgiveness misunderstands both forgiveness and courage. One can be required to act courageously. And while forgiveness resembles courage—and may sometimes be a courageous act—it also resembles honesty, temperance, and wisdom. Forgiveness is helpfully understood as a virtue, but it is not at all clear which one, or which type, it most resembles.

But, if one is sometimes morally required to act virtuously, and forgiveness is like other virtues in this respect, then one might sometimes be morally required to forgive (Murphy 1982; Richards 1988; Griswold 2007). Moreover, if part of being virtuous is perceiving the world as one has most reason to, and if it is sometimes irrational to fail to perceive the world as it presents itself, then failure to forgive when the world presents an offender as worthy of forgiveness can be irrational in the same way. For example, one feature of cowardice is being fearful beyond what’s warranted. Sometimes one’s excessive fear is even irrational, as when one perceives a minor risk as a mortal danger. An unwillingness or inability to recognise that one no longer has any good reason to blame—beyond the fact of culpable wrongdoing—seems irrational in the same way.

## Conclusion

I have argued that forgiveness is not essentially elective. Not only have its proponents not made an adequate case for EEF, but its intuitive plausibility diminishes upon reflection. Any defense of EEF must explain why reasons to forgive cannot generate requirements while other similar reasons can. Nonetheless, I don’t deny that forgiveness is often morally and rationally optional. As is often the case when deciding how to act, we do not always know what we have most reason to do. And the reasons for and against forgiving may not always be decisive. However, forgiveness can, in principle, be required for any offence. Whether and when forgiveness is elective depends on contingent facts about the offence, the offender, and potential forgiver. There is, of course, more to be said about elective forgiveness, but I hope at least to have illuminated the structure of the debate and the considerations that must be addressed by any adequate account of the optionality of forgiveness.

## Footnotes

1 John Martin Fischer and Mark Ravizza explain acting for reasons without being aware of those reasons (1998, 64 and 85–87). See also Robert Audi (1986).

2 See also Pamela Hieronymi (2001, 552). Recognition of this tension can also be found in the historical debates about the nature and purpose of the Catholic sacrament of penance, which address many of the same points (Martos 1981).

3 Eve Garrard and David McNaughton (2003, 2010) argue that unconditional forgiveness—e.g., forgiveness of unrepentant wrongdoers—is always morally permissible. However, this is not a strongly elective view of the sort I am addressing in this paper. Their view is compatible with my claim that forgiveness *can be* and is sometimes required. Indeed, they object to accounts, like some strongly elective views, which treat the forgiven as undeserving of forgiveness, describing such accounts as a “thin and etiolated version of forgiveness” (2003, 58). Their view is also technically compatible with my claim that forgiveness *can be* impermissible. They argue that a particular reason for forgiving—i.e. solidarity/love (2010, 120)—is, *as a matter of fact*, always sufficient to render forgiveness permissible. I discuss this claim in Section 4.

4 Some suggest that forgiveness does not require an emotional change (Nelkin 2013; Warmke 2016a) and others that it doesn’t require a change of attitude at all (Cornell 2017). For example, forgiveness may only require that one act in a way that changes the normative relations between forgiver and offender (Warmke 2016b). However, even on this kind of view, whether one could be required to forgive is an open question. Moreover, these views are incompatible with some of the most plausible defences of elective forgiveness (see Section 4.3 below).

5 Dana Nelkin (2013) is an exception.

6 Or consider individuals whose blame is likely to be ignored or devalued because of their social position. The poet Claudia Rankine describes how people of colour must often “move on” or “let go” of their blame in order to overcome the debilitating effects of everyday racism (2014, 79). However, she does not (I think) conceive of this phenomenon as forgiveness. These examples do not show that overcoming blame for prudential reasons is never forgiveness. Barrett Emerick (2017), following Kathryn Norlock (2008), suggests that it can be. I disagree, but for now it is enough to note that both philosophy and common usage seem to recognise ways of overcoming blame that are not forgiving because they are done for the wrong reasons.

7 J. Angelo Corlett states that the idea of a requirement to forgive is “a queer and cruel irony of justice” (2006, 36), but gives no argument for this claim. Howard McGary (1989) gives a very brief argument for EEF, which I address below (Section 4.4). Alternatively, some might hold that forgiveness can be morally but not rationally required. Brandon Warmke appears to defend such a position (2015, 498). I consider his view below (Section 4).

8 Pettigrove could commit himself to EEF by arguing that non-gracious forgiveness is not genuine forgiveness. However, this position would be subject to the same concerns I raise later against Calhoun. Alternatively, he could embrace pluralism about forgiveness. Perhaps there are different types, each with a particular purpose and set of conditions (Bennett 2003, 129; Nelkin 2011, 47) or perhaps some circumstances call for “imperfect forgiveness” (MacLachlan 2009). If so, the tension between gracious and non-gracious forgiveness might be understood as the confusion of these two conceptions and their associated practices. Pettigrove himself is content simply to note that forgiveness is a “diverse phenomenon” (2012, 159) and I will do the same. The pluralism question must wait for another time.

9 An agent might give extra weight to the reasons she thinks are most important. For example, I might have all things considered most reason to donate my extra income to a maximally effective charity, but nonetheless be permitted to spontaneously buy my nephew a present. However, even if we accept a prerogative of this sort, one’s reasons to forgive might still generate a requirement. They might outweigh not only the competing reasons but also the prerogative, just as my prerogative to spend some of my extra income on my nephew can be overwhelmed by other considerations. Thus, depending on its strength, a prerogative might secure *frequently elective forgiveness*, but not essentially elective forgiveness.

10 This is the main challenge facing Garrard and McNaughton’s defence of unconditional forgiveness (2003, 2010). While they offer a compelling explanation of why solidarity/love is a good reason to cease blaming, they simply assume that this reason is never overridden. (Neither their paper (2003) nor their book takes up the question raised above.) But this claim requires argument. Garrard and McNaughton must show that solidarity/love is always sufficient to render forgiveness permissible—i.e. that it always outweighs any and all reasons against forgiving.

11 Calhoun may now view forgiveness as an example of what she has since defined as “common decency,” namely, actions that, “we expect any minimally well-formed agent will *elect* to do for others *absent any requirement* to do so” (2004, 130; emphasis in original). Such acts are nonobligatory, but also not fully morally elective. However, whether she views forgiveness as essentially elective or as a common decency—or, for that matter, as a simple favour (Feinberg 1961), a suberogatory action (Driver 1992), or a morally permissible moral mistake (Harman 2016)—her argument for AF will presumably provide the justification for that position. Moreover, my response will apply regardless because I argue that forgiveness is not essentially elective *and* that it’s (sometimes) impermissible to forgive (or not).

12 On this view, the main purpose of blame is as a form of moral address, demand, or protest (Hieronymi 2001, McKenna 2012, or Smith 2013), including forms of political rather than moral address (MacLachlan 2010).

13 One might challenge the argument further. First, it is not obvious that reasons to forgive are always reasons to feel general benevolence either. Forgiving may be inseparable from such feelings, but they do not define forgiveness anymore than they define gratitude or admiration, which are also plausibly inseparable from feelings of general benevolence. Second, one might contest the claim that reasons for or against romantic love cannot generate requirements. For example, Abramson and Leite argue that one can be required *not* to love another. They imagine a couple, Jamie and Morgan, in which one partner has “gradually come to be manipulative, selfish, insulting and occasionally cruel.” And they suggest that, while Jamie may continue have strong reasons to try to prevent Morgan from becoming a lost cause, “in the face of ongoing significant hurtful behaviour […], continued love can be appropriate only so long as there is a realistic hope of relevant improvement” (2011, 688–689). These arguments, if compelling, provide additional reasons, beyond the dilemma raised above, to reject the love defense of EEF.
